# Comparative Study on the Interaction Between Micelles of Gemini Quaternary Ammonium Surfactants with Different Counterions and Curcumin

**DOI:** 10.3390/foods15213972

**Published:** 2026-11-05

**Authors:** Siqi Hu, Runyu Cao, Xinyue Tai, Peng Xu, Jizhou Zhu, Xing Yan, Hongqin Liu, Mingquan Huang

**Affiliations:** 1 Jiangxi Province Key Laboratory of Sustainable Utilization of Traditional Chinese Medicine Resources, Institute of Traditional Chinese Medicine Health Industry, China Academy of Chinese Medical Sciences, Nanchang 330115, China; hsqjks@163.com (S.H.);; 2 Key Laboratory of Brewing Molecular Engineering of China Light Industry, Beijing Technology & Business University, Beijing 100048, China; 3 Jiangxi Shunfutang Traditional Chinese Medicine Decoction Pieces Co., Ltd. Nanchang 331409, China

**Keywords:** curcumin, gemini quaternary ammonium surfactants, counterion effect, micelle, antioxidant activity

## Abstract

Curcumin is a natural polyphenol with diverse pharmacological activities, but its poor aqueous solubility and rapid alkaline degradation severely limit its application. In this study, a series of gemini quaternary ammonium surfactants with different counterions, 12-2-12·2Y (Y = CH_3_CO_3_^−^, CH_3_COO^−^, CH_3_CH(OH)COO^−^, CH_3_(CH_2_)_3_COO^−^, C_6_H_5_COO^−^, and Br^−^, abbreviated as Mc, Ac, Lac, Va, Bz and Br), were used to prepare micellar systems for curcumin solubilization and stabilization. The interaction between the trianionic form of curcumin (Cur^3−^) and the micelles were investigated. It was demonstrated that Cur^3−^ was preferentially distributed in the palisade layer via electrostatic attraction to the headgroups of 12-2-12·2Y. The type of counterion significantly affected the micropolarity of the palisade layer and the fluorescence quantum yield of Cur^3−^. Cur^3−^ exhibited the highest quantum yield (1.70 × 10^−3^) in the 12-2-12·2 Va micelles, which may be due to their highly compact and hydrophobic palisade layer, whereas the lowest yield (1.79 × 10^−4^) was obtained in the 12-2-12·2Br micelles, possibly due to the strong hydration of Br^−^ and its tight adsorption onto the headgroups. After 24 h at pH 13, Cur^3−^ retention was 54.30–82.29% in the micellar systems, compared with only 4.21% in water. DPPH radical scavenging assays demonstrated that micellar solubilization/stabilization can enhance the antioxidant activity of curcumin. Curcumin exhibited the highest DPPH scavenging activity in the 12-2-12·2Bz system and the lowest in the 12-2-12·2Mc system. These results revealed that counterions may regulate the positioning, fluorescence behavior, chemical stability and antioxidant activity of Cur^3−^ in the palisade layer via multiple mechanisms, including electrostatic attraction/repulsion, the balance between hydrophilic and hydrophobic forces, and steric hindrance. Practical translation will require pH-compatible, food-grade surfactant systems and dedicated toxicological evaluation.

## 1. Introduction

Curcumin, the main bioactive ingredient in turmeric extract, has a long history of use as an important ingredient in food products. In recent years, curcumin has attracted considerable research attention owing to its beneficial effects on human health, including antioxidant, anti-inflammatory, antitumor, antibacterial, and neuroprotective effects [1,2,3]. However, curcumin is extremely poorly soluble in water, which reduces its bioavailability. Furthermore, due to the instability of its β-diketone group, curcumin rapidly degrades in alkaline environments. These two factors severely limit its applications [2,4]. Various strategies have been developed to overcome the poor water solubility and low bioavailability of curcumin. These methods include reducing particle size and forming complexes with cyclodextrins, which enhance solubility by increasing the specific surface area and forming water-soluble inclusion complexes [5,6,7]. Other strategies involve encapsulating curcumin in colloidal carrier systems such as nano emulsions, liposomes and solid lipid nanoparticles [8,9,10], or converting crystalline curcumin into an amorphous state [11].

Due to their unique core–shell structure, surfactant micelles can encapsulate curcumin molecules through various weak interactions, including hydrophobic, electrostatic and hydrogen bonds. This significantly enhances the solubility, stability and bioavailability of curcumin [12]. Among the various surfactant systems, gemini surfactants—composed of two hydrophobic tails and two hydrophilic headgroups covalently linked by a spacer—have lower critical micelle concentrations (CMCs), greater surface activity, and improved aggregation properties compared to conventional single-chain surfactants [13]. Previous studies have demonstrated that cationic gemini surfactant micelles can effectively encapsulate curcumin and improve its stability. Wang et al. systematically investigated the interactions between curcumin and various cationic surfactants, including trimeric, gemini and monomeric surfactants. They found that gemini surfactant is the best choice to effectively suppress Cur^3−^ degradation at very low concentrations [14]. Ke et al. reported that the spacer length of gemini surfactants alkanediyl-α, ω-bis (dodecyldimethylammonium bromide) (C_12_C*_s_*C_12_Br_2_, where *s* = 2, 3, 4, 6, and 12, indicating the number of carbons in the spacer) significantly affects the electrostatic interactions between its micelles and Cur^3−^, with maximum fluorescence intensity observed at a spacer length of s = 6 [15]. Anjudikkal et al. found that the solubilization ability of short-spacer gemini surfactant on curcumin was higher than long-spacer gemini surfactant, while the stabilization order was reversed [2]. Furthermore, Kumar et al. found that curcumin was distributed in the palisade layer of mixed surfactant aggregates composed of equimolar double- and single-chain mixed ionic surfactants [16].

It is worth noting that the type of counterions in surfactants has a significant impact on the interface properties of micelles. The counterions regulate the surface charge density, hydrophilic–hydrophobic balance, and steric hindrance of the micelles, thereby controlling the microstructure of the micelles and interactions with the guest molecules [17]. However, there is a lack of systematic studies on how counterions affect the interaction between gemini quaternary ammonium surfactant micelles and Cur^3−^, especially regarding the regulation of the palisade-layer microenvironment and its consequent effects on the fluorescence properties, stability, and antioxidant activity of Cur^3−^.

In this work, a series of gemini quaternary ammonium surfactants with different counterions were synthesized according to our previous work [18,19]. The formula was C_11_H_23_CONH(CH_2_)_2_N^+^(CH_3_)_2_(CH_2_)_2_(CH_3_)_2_N^+^(CH_2_)_2_NHCOC_11_H_23_·2Y (Y = CH_3_CO_3_^−^, CH_3_ COO^−^, CH_3_CH(OH)COO^−^, CH_3_(CH_2_)_3_COO^−^, C_6_H_5_COO^−^, Br^−^). The interactions between Cur^3−^ and the surfactant 12-2-12·2Y micelles were systematically studied. It should be emphasized that synthetic cationic gemini quaternary ammonium surfactants are not generally approved as direct food additives or food-contact materials and may exhibit cytotoxicity. Additionally, the strongly alkaline conditions used in this study do not reflect those of most foods or physiological environments. Therefore, the aim of this study is to reveal the molecular mechanisms by which the counterions of gemini quaternary ammonium surfactants regulate the micellar interfacial microenvironment, and the subsequent effects on the fluorescence behavior, chemical stability and antioxidant activity of Cur^3−^, rather than to propose an immediate food formulation.

## 2. Experiment

### 2.1. Materials

Gemini quaternary ammonium surfactants with different counterions, 12-2-12·2Y, were synthesized according to our previous work [18,19]. Curcumin and coumarin 153 were analytical grade and purchased from Sigma-Aldrich (Shanghai, China) Trading Co., Ltd. Pyrene was provided by Beijing MREDA Technology Co., Ltd. (Beijing, China). Methanol was analytical grade and purchased from Sinopharm Chemical Reagent Co., Ltd. (Shanghai, China). The ultrapure water was obtained by a Milli-Q ultrapure (Merck, Darmstadt, Germany) water purification system.

### 2.2. Determination of CMC and Micellar Micropolarity by Pyrene Fluorescence Method

A FLS100 fluorescence spectrometer (Edinburgh Instruments, Livingston, UK) was employed to determine the CMC and the micropolarity of the surfactant 12-2-12·2Y^−^, using pyrene as a fluorescent probe. A series of surfactant solutions with concentrations ranging from 1 × 10^−7^ to 0.01 mol/L were prepared using a saturated aqueous pyrene solution at a concentration of 1 × 10^−7^ mol/L as the solvent. The fluorescence emission spectra were recorded at 298.15 K, with an excitation wavelength of 335 nm. The excitation and emission slit widths were set to 5 nm and 2.5 nm, respectively, and spectra were scanned from 350 to 600 nm at a speed of 60 nm/min. The fluorescence intensities of pyrene at peaks *λ*_1_ (373 nm) and *λ*_3_ (384 nm), i.e., *I*_1_ and *I*_3_, were recorded from the emission spectrum at each concentration. Each sample was measured three times. The fluorescence intensity was the average of the three measurements, with a standard deviation of less than 0.002.

### 2.3. Steady-State Fluorescence Measurement

All surfactant solutions were prepared in ultrapure water at a concentration four times the CMC to ensure the formation of micelles. A 5 mg/mL solution of curcumin in methanol was added to either ultrapure water or the micellar solution, achieving a final Cur^0^ concentration of 40 μM. To prepare the Cur^3−^ solution, a specific volume of 4 M NaOH was added to the Cur^0^ solution until the resulting pH was 13. The fluorescence emission spectra of Cur^0^ in ultrapure water and micellar solutions were recorded from 430 to 750 nm, with an excitation wavelength of 420 nm, whereas the spectra of Cur^3−^ were acquired from 500 to 800 nm, with an excitation wavelength of 460 nm.

### 2.4. Determination of Fluorescence Quantum Yield

The fluorescence quantum yields of Cur^3−^ in ultrapure water and 12-2-12·2Y micellar solutions were measured using an F4600 fluorescence spectrophotometer (Hitachi High-Tech Corporation, Tokyo, Japan), with coumarin 153 employed as a secondary standard. The absolute quantum yield of coumarin 153 in acetonitrile at 298.15 K is 0.56 [3]. The quantum yield of Cur^3−^ was then calculated using the following equation [3,20],

(1) $$\frac{\Phi_{S}}{\Phi_{R}}=\frac{A_{S}}{A_{R}}\frac{{\left(\mathrm{Abs}\right)}_{R}}{{\left(\mathrm{Abs}\right)}_{S}}\frac{{n_{S}}^{2}}{{n_{R}}^{2}}$$

where Φ is the fluorescence quantum yield, A is the integrated area under the fluorescence emission spectrum, Abs is the absorbance at the excitation wavelength, and n is the refractive index of the solvent (1.33 for water and 1.34 for acetonitrile). The subscripts S and R refer to the sample and the reference, respectively.

### 2.5. Cu^2+^-Induced Fluorescence Quenching Assay

Cu^2+^ is a typical ionic fluorescence quencher that is primarily distributed in the aqueous phase and the palisade layer of micelles rather than in the hydrophobic core. Therefore, the extent of fluorescence quenching of Cur^3−^ by Cu^2+^ can be used to infer the binding site of Cur^3−^ within the micelles. The concentration of 12-2-12·2Y was fixed at 4 CMC. The concentration of Cu^2+^ ranged from 0.3 to 2.7 μM. The fluorescence conditions were the same as those used in the steady-state fluorescence experiment described above. The quenching of Cur^3−^ by Cu^2+^ follows the Stern–Volmer equation [21],

(2) $$\frac{F_{0}}{F}=1+K\mathrm{sv}\left[Q\right]$$

where *F*_0_ and *F* are the fluorescence emission intensities of Cur^3−^ in the absence and presence of Cu^2+^, respectively; [*Q*] is the concentration of Cu^2+^; and *K*_SV_ is the Stern–Volmer quenching constant, obtained from the slope of the linear plot of *F*_0_/*F* versus [*Q*].

### 2.6. UV-Vis Absorption Measurement

The UV-vis absorption spectra of Cur^3−^ (40 μM) in ultrapure water and 12-2-12·2Y micellar solutions (4 CMC) were recorded using a Shimadzu UV 3600 UV-vis spectrophotometer at 298.15 K. The absorbance spectra were recorded every 3 h over a total period of 24 h. The retention rate of Cur^3−^ was calculated based on the decrease in absorbance at 460 nm as a function of time [3]. This approach assumes that the apparent molar absorptivity of Cur^3−^ at 460 nm remains constant within each medium over the 24 h degradation period. As all systems have the same initial concentration of Cur^3−^ and detection wavelength, this assumption does not affect the relative comparison of degradation rates among different surfactant micelle systems.

### 2.7. ^1^H NMR Spectra Measurement

The ^1^H NMR spectra of surfactants 12-2-12·2Y before and after the addition of curcumin were acquired on a Bruker AV-300 NMR spectrometer (Bruker Corporation, Billerica, MA, USA) using CDCl_3_ as the solvent.

These measurements are intended to probe specific non-covalent interactions in a non-aqueous environment and are not a direct model of the aqueous micellar interface.

### 2.8. DPPH Radical Scavenging Ability Measurement

A 0.2 mmol/L ethanolic solution of DPPH was first prepared as the radical source. Subsequently, a series of aqueous solutions were prepared, comprising DPPH alone, DPPH with curcumin, and DPPH with curcumin combined with varying concentrations of the gemini quaternary ammonium surfactant 12-2-12·2Y. The concentration of curcumin was fixed at 0.8 µM, while the concentration of 12-2-12·2Y was fixed at 4 CMC. All solutions were kept in the dark for 30 min, after which the absorbance of DPPH at its maximum absorption wavelength of 517 nm was recorded using a Shimadzu UV-3600 UV-vis spectrophotometer (Shimadzu Corporation, Kyoto, Japan).

## 3. Results and Discussion

### 3.1. Micellization Behavior of Surfactants 12-2-12·2Y

**Figure 1:**
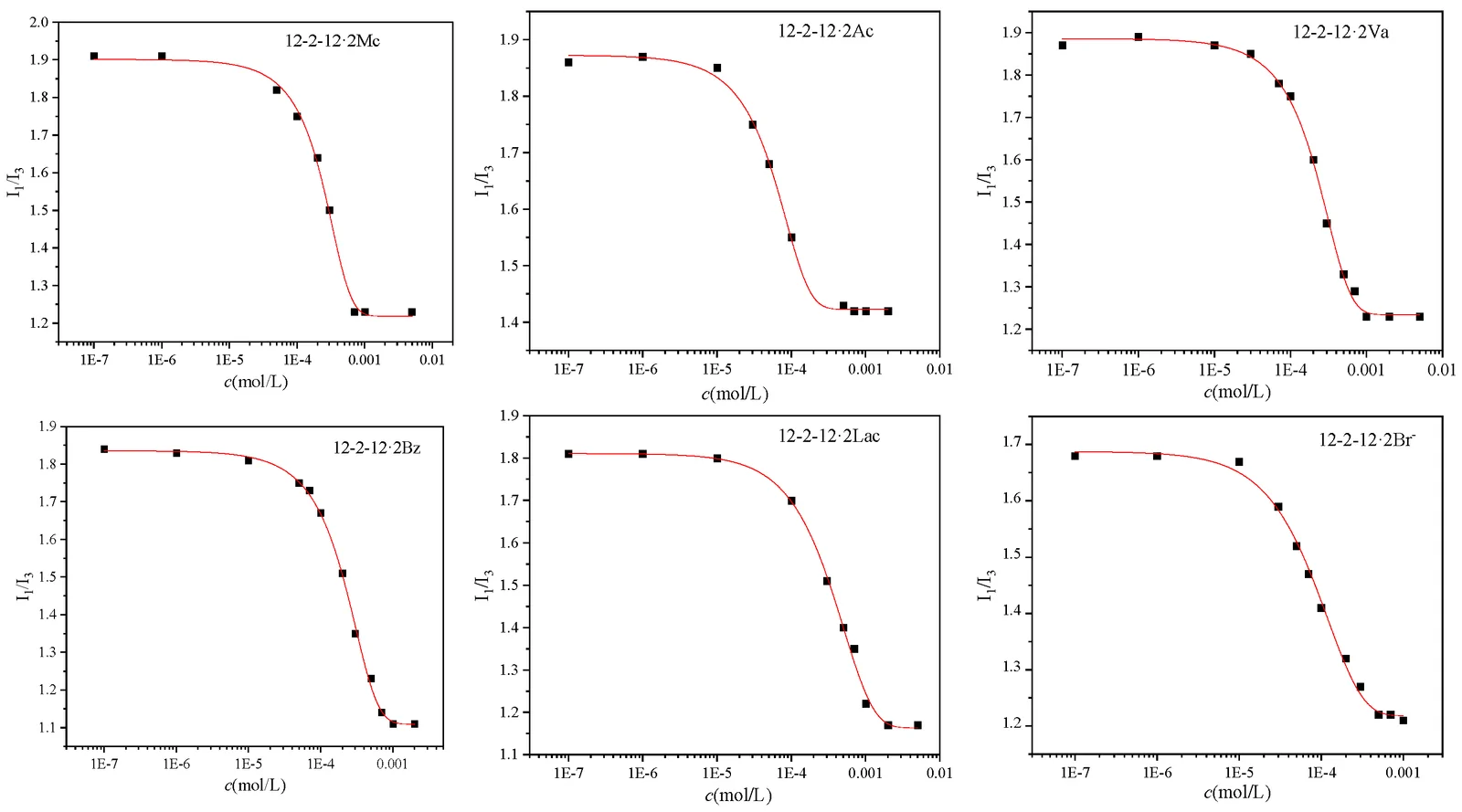
Variation in *I*_1_/*I*_3_ with the concentration (*c*) of surfactants 12-2-12·2Y.

The CMC of the gemini quaternary ammonium surfactants 12-2-12·2Y was determined using the pyrene fluorescence probe method. Figure 1 shows the variations in pyrene polarity ratio (*I*_1_/*I*_3_) versus surfactant concentration. The CMC values of the surfactants, which are determined by the point at which the initial and final horizontal asymptotes of the S-shaped curve in Figure 1 intersect, are listed in Table 1. The *I*_1_/*I*_3_ ratios at final horizontal asymptotes of the curves, which reflect the final micropolarity of the surfactant aggregates, are also listed in Table 1 [22].

As shown in Table 1, these gemini quaternary ammonium surfactants exhibit excellent surface activity, with CMC values ranging from 7.57 × 10^−5^ to 2.56 × 10^−4^ mol/L, significantly lower than those of corresponding single-chain quaternary ammonium surfactants due to the enhanced hydrophobic effect of the two hydrophobic tails in gemini molecules [12,23]. Table 1 also shows that the order of the CMC values of these surfactants is as follows: 12-2-12·2Mc >12-2-12·2Va >12-2-12·2Br > 12-2-12·2Lac > 12-2-12·2Bz > 12-2-12·2Ac. The effect of counterions on the CMC is a complex process involving hydrophobic effects, steric hindrance, electrostatic interactions, hydrogen bonding and other factors [17]. As the length of the hydrophobic carbon chain of the counterion increases, the overall hydrophobicity of the surfactant molecule increases, making it easier for the molecules to aggregate and lowering the CMC. Therefore, surfactant 12-2-12·2Ac has a lower CMC. However, the CMC of 12-2-12·2Va actually increases. This is because the further increase in the counterion chain length leads to excessive steric hindrance at the micellar interface, which disrupts the tight, ordered packing of monomers within the micelle core, resulting in an increase in CMC [24]. In addition, for surfactant 12-2-12·2Lac, the -OH in CH_3_CH(OH)COO^−^ can form intermolecular hydrogen bonds, thereby enhancing its hydrophilicity and resulting in a higher CMC [25]. For surfactant 12-2-12·2Bz, the rigid benzene ring of C_6_H_5_COO^−^ may promote micellization through strong hydrophobic effects and π–π stacking interactions, resulting in a low CMC [26].

Table 1 also clearly demonstrates the significant regulatory role of the counterion microenvironment on surfactant aggregation. Notably, 12-2-12·2Ac exhibits the lowest CMC but the highest I_1_/I_3_ (1.42), suggesting that its micellar structure is relatively loose and the solubilization site of pyrene is exposed to a more polar environment. Conversely, 12-2-12·2Bz shows the lowest I_1_/I_3_ (1.11), suggesting a highly compact hydrophobic core with extremely low polarity [27]. These microenvironmental differences may underlie the subsequent differences in the binding behavior and stability of curcumin in different micelles.

### 3.2. Steady-State Fluorescence Spectra

**Figure 2:**
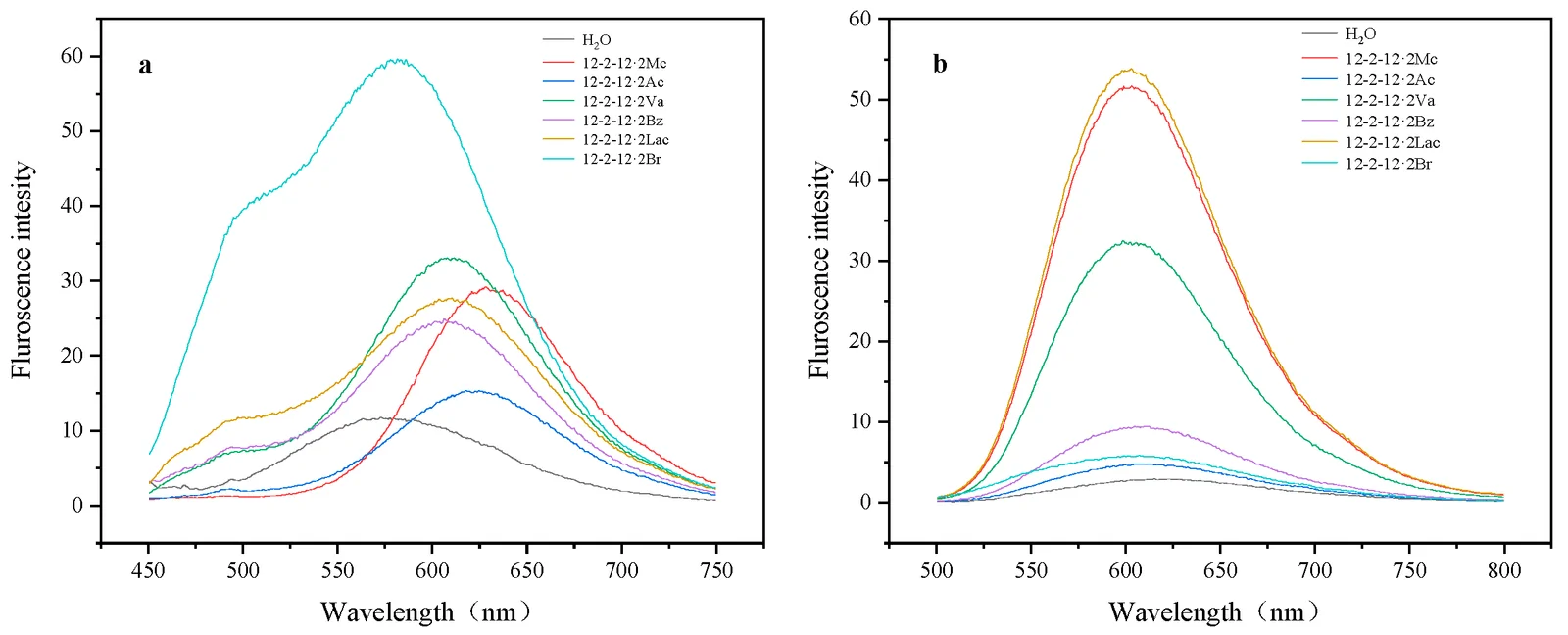
Steady-state fluorescence spectra of Cur^0^ ((**a**), pH = 7) and Cur^3−^ ((**b**), pH = 13) in ultrapure water and 12-2-12·2Y micellar solutions at 298.15 K.

At pH 7, curcumin predominantly exists as the neutral form (Cur^0^), while at pH 13, the three acidic protons of the enolic and phenolic hydroxyl groups are completely dissociated, resulting in the formation of the fully deprotonated trianion (Cur^3−^) [28]. Figure 2 depicts the steady-state fluorescence spectra of Cur^0^ and Cur^3−^ in ultrapure water and surfactant micellar solutions. As illustrated in Figure 2a, at pH 7, Cur^0^ exhibits weak fluorescence in ultrapure water, characterized by a broad emission band centered around 560 nm. However, the fluorescence intensity in the 12-2-12·2Y micelles is significantly enhanced by two- to six-fold, accompanied by an evident red shift, suggesting that Cur^0^ transfers from the aqueous phase into the hydrophobic core of a micelle, driven by hydrophobic interactions [29]. Meanwhile, the ordered micellar structure restricts the rotation of curcumin molecules and induces their conformational planarization, resulting in a red-shifted emission [30]. On the other hand, at pH 13, Cur^3−^ displays an emission spectrum centered around 610 nm in ultrapure water. However, in the 12-2-12·2Y micelles, the fluorescence intensity significantly increases, accompanied by a weak blue shift. This phenomenon indicates that there is strong electrostatic attraction between the negatively charged Cur^3−^ and the positively charged headgroups of the 12-2-12·2Y micellar structure, causing Cur^3−^ to be preferentially distributed in the palisade layer of the micelles [12]. It is known that the palisade layer of micelles mainly consists of headgroups, counterions, an amide group and adjacent α-methylene groups of alkyl chains, spacers, and some water molecules, which leads to weak blue shift of Cur^3−^ in the palisade layer compared to that of Cur^3−^ in water [31].

### 3.3. Fluorescent Quantum Yield

Using coumarin 153 as the reference, the fluorescent quantum yields of Cur^3−^ in ultrapure water and 12-2-12·2Y micellar solutions were calculated according to Equation (1). The results are summarized in Table 2. In general, the stronger a fluorescent probe binds to water, the lower its fluorescence quantum yield [32].

As shown in Table 2, the fluorescence quantum yield of Cur^3−^ in the 12-2-12·2Br^−^ micelles is close to that in ultrapure water, indicating that Cur^3−^ does not effectively enter the micellar interior. In contrast, the fluorescence quantum yield increases to varying degrees in the other surfactant micelles, suggesting that these micelles can sequester Cur^3−^ effectively from the aqueous phase. This blocks the interaction between Cur^3−^ and water molecules, consequently enhancing fluorescence efficiency. Notably, the fluorescence quantum yield of Cur^3−^ in the 12-2-12·2Va micelles is approximately eight times that in ultrapure water. CH_3_(CH_2_)_3_COO^−^ possesses the longest hydrophobic chain, which may render the palisade layer most compact and hydrophobic. Cur^3−^ embedded therein has minimal contact with water, thus affording the highest quantum yield. Cur^3−^ also exhibits relatively high fluorescence quantum yields in the 12-2-12·2Mc and 12-2-12·2Bz micelles. This may be because CH_3_CO_3_^−^ and C_6_H_5_COO^−^ interact only weakly with water and are readily ionized, thereby promoting stronger electrostatic attraction between the headgroups of 12-2-12·2Mc and 12-2-12·2Bz and Cur^3−^ [33]. In addition, the phenyl ring of C_6_H_5_COO^−^ may enhance the structural order of the micelles through π–π stacking interactions, providing a relatively ordered microenvironment for Cur^3−^. The fluorescence quantum yield of Cur^3−^ in the 12-2-12·2CH_3_CO_3_^−^ micelles is relatively low, possibly due to its relatively loose micellar structure. In contrast, the fluorescence quantum yield of Cur^3−^ in the 12-2-12·2Br micelles is extremely low. This may be due to the strong hydrophilicity and high charge density of Br^−^, enabling it to form a robust hydration layer while also adsorbing tightly onto the positively charged surfactant headgroups [34]. This partially neutralizes the positive charge of the headgroups, weakening the electrostatic attraction to Cur^3−^ and creating a repulsive barrier at the micellar surface simultaneously. Consequently, Cur^3−^ cannot effectively enter the palisade layer and remains largely exposed to the aqueous phase, resulting in a fluorescence quantum yield comparable to that in ultrapure water.

### 3.4. Cu^2+^-Induced Fluorescence Quenching

**Figure 3:**
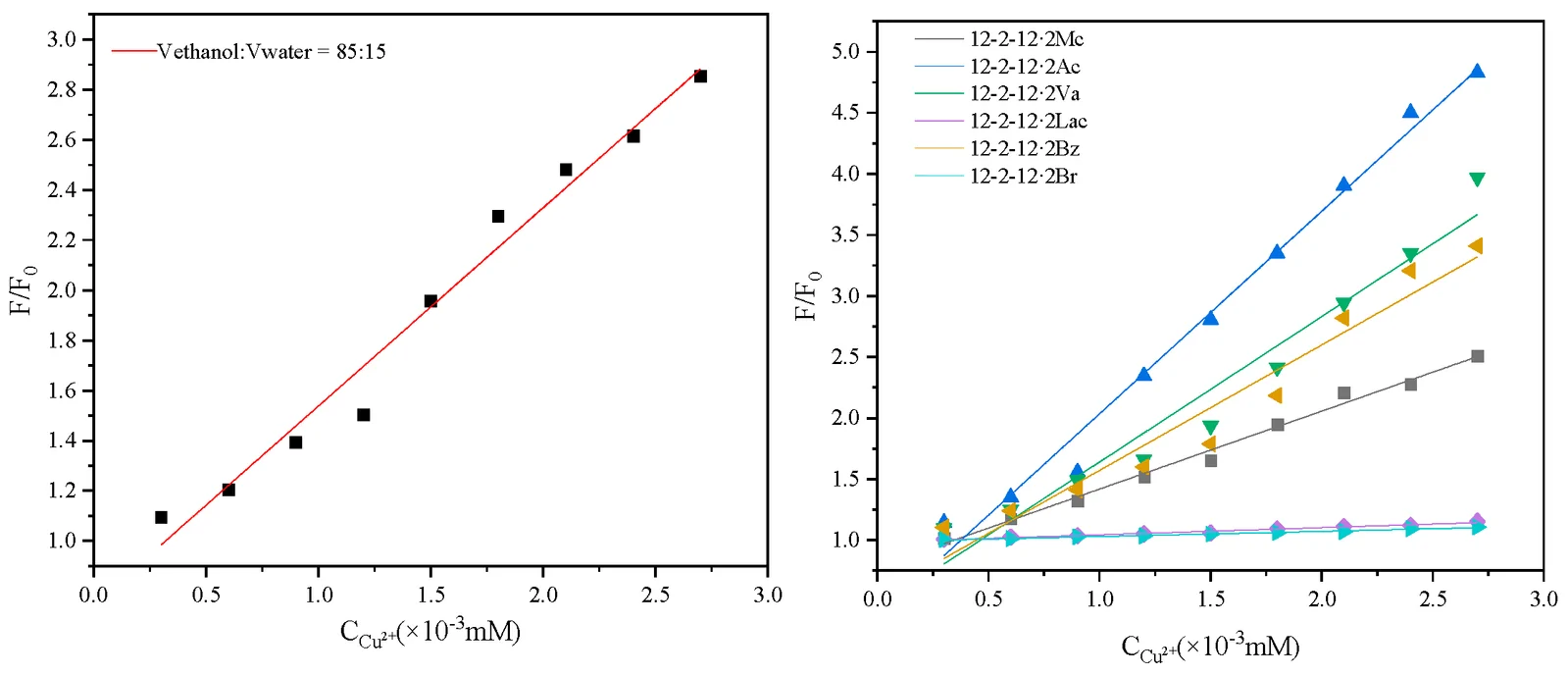
The Stern–Volmer plots for Cu^2+^-induced fluorescence quenching in the solvent (V_ethanol_:V_water_ = 85:15) and 12-2-12·2Y micelle solutions.

Cu^2+^ quenching experiments further revealed the binding sites of curcumin. As a typical ionic quencher, Cu^2+^ mainly distributes in the aqueous phase and the palisade layer of the micelles, rather than in the hydrophobic core [35]. Figure 3 shows the Stern–Volmer plots for Cu^2+^-induced fluorescence quenching in the solvent (V_ethanol_:V_water_ = 85:15) and 12-2-12·2Y micelle solutions. The slope of each plot gives the Stern–Volmer quenching constant (*K*_SV_), which is listed in Table 3. The higher the slope, the more efficient the quenching.

As shown in Table 3, the *K*_SV_ values of the 12-2-12·2Ac, 12-2-12·2Va and 12-2-12·2Bz systems are greater than that of the solvent (V_ethanol_:V_water_ = 85:15), indicating that Cur^3−^ is preferentially distributed in the palisade layer of these surfactant micelles. In contrast, the *K*_SV_ values of the 12-2-12·2Lac system are much lower than that of the solvent. This may be because the strong hydrophilicity of CH_3_CH(OH)COO^−^ enables it to form a thick hydration layer on the barrier-layer surface, thereby hindering the penetration of Cu^2+^ [36]. The resulting hydration barrier is thinner since the polarity of CH_3_CO_3_^−^ is slightly weaker than that of CH_3_CH(OH)COO^−^, resulting in a *K*_SV_ value that is slightly higher than that of the 12-2-12·2CH_3_CH(OH)COO^−^ system but lower than that in the solvent. In addition, as described in Section 3.3, Cur^3−^ is primarily exposed to the aqueous phase in the 12-2-12·2Br system. However, due to the high charge density of Br^−^, the tightly adsorbed layer formed on the positively charged headgroups simultaneously repels Cu^2+^, resulting in a lower *K*_SV_ value [37].

### 3.5. Degradation Rate

**Figure 4:**
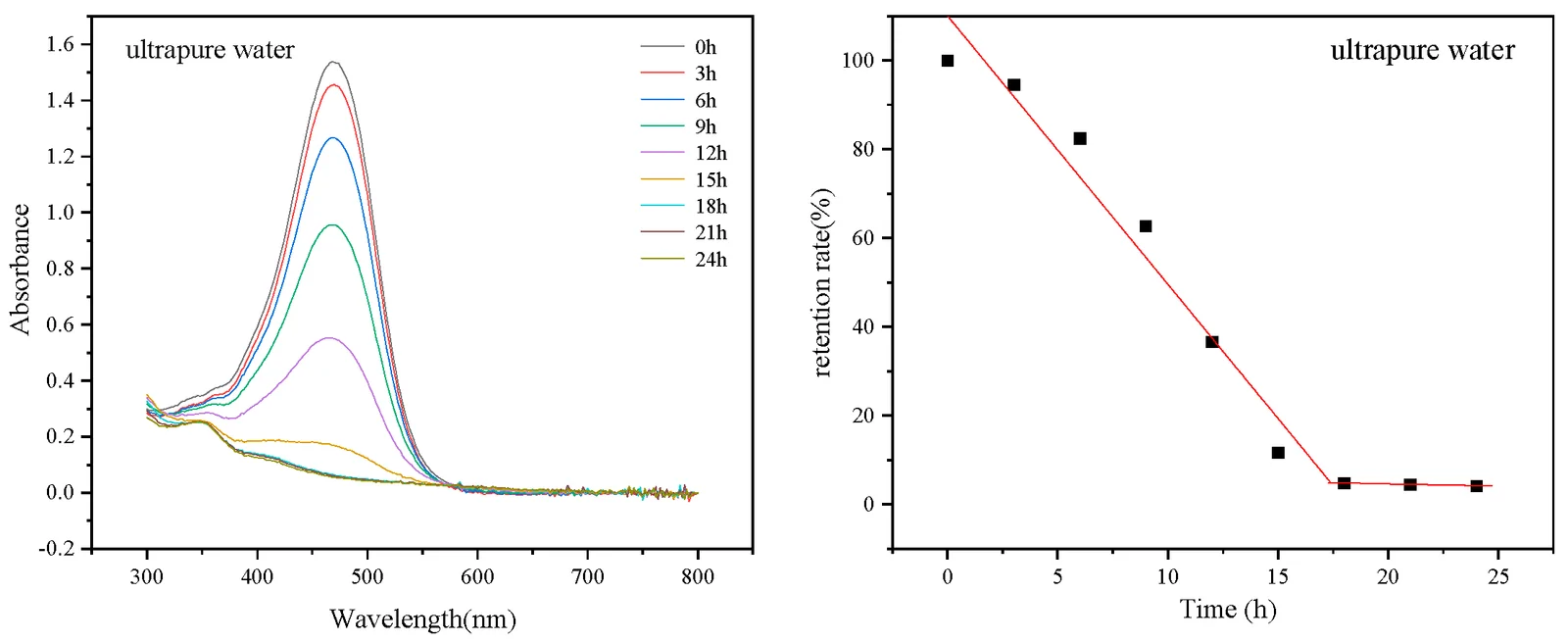

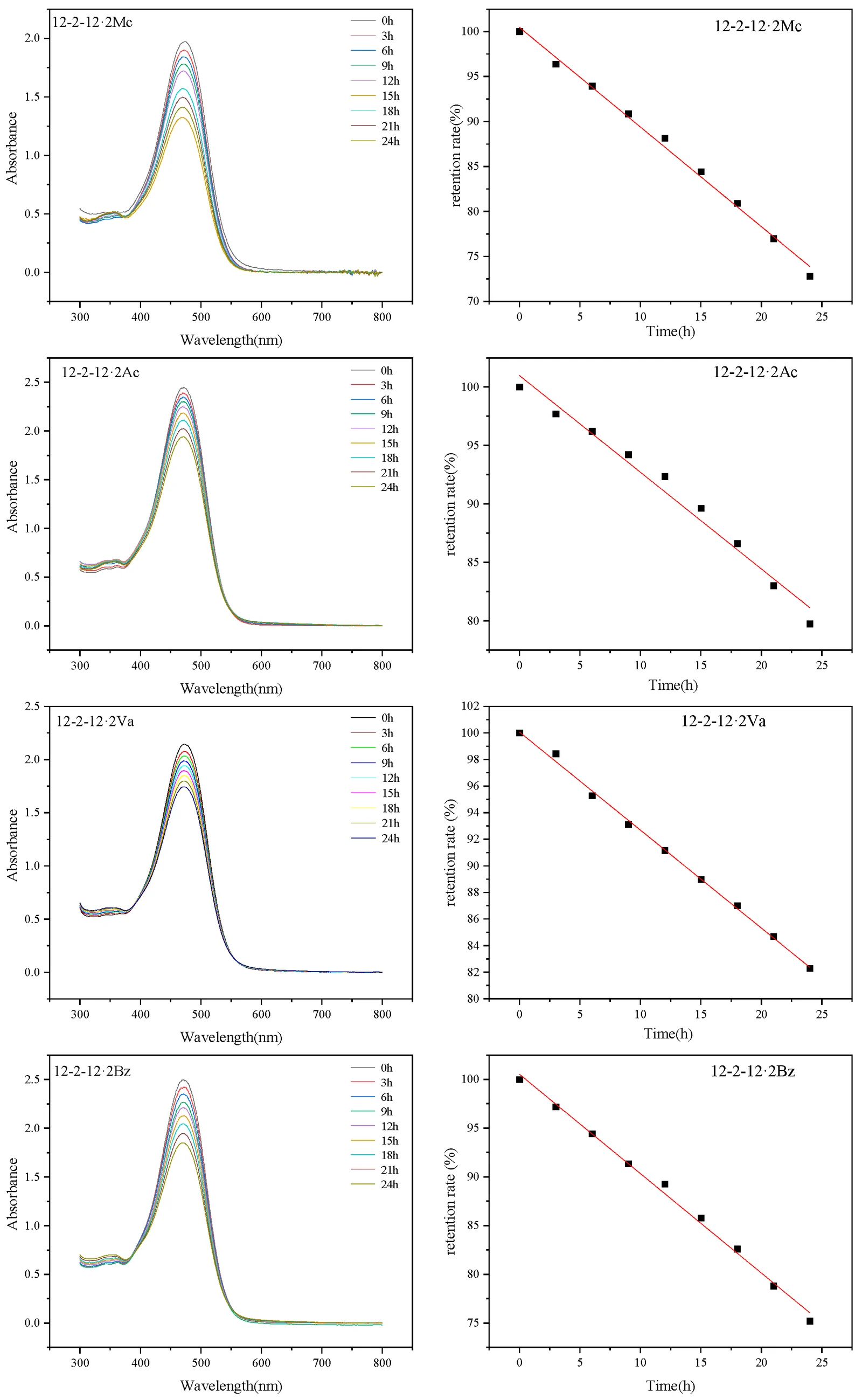

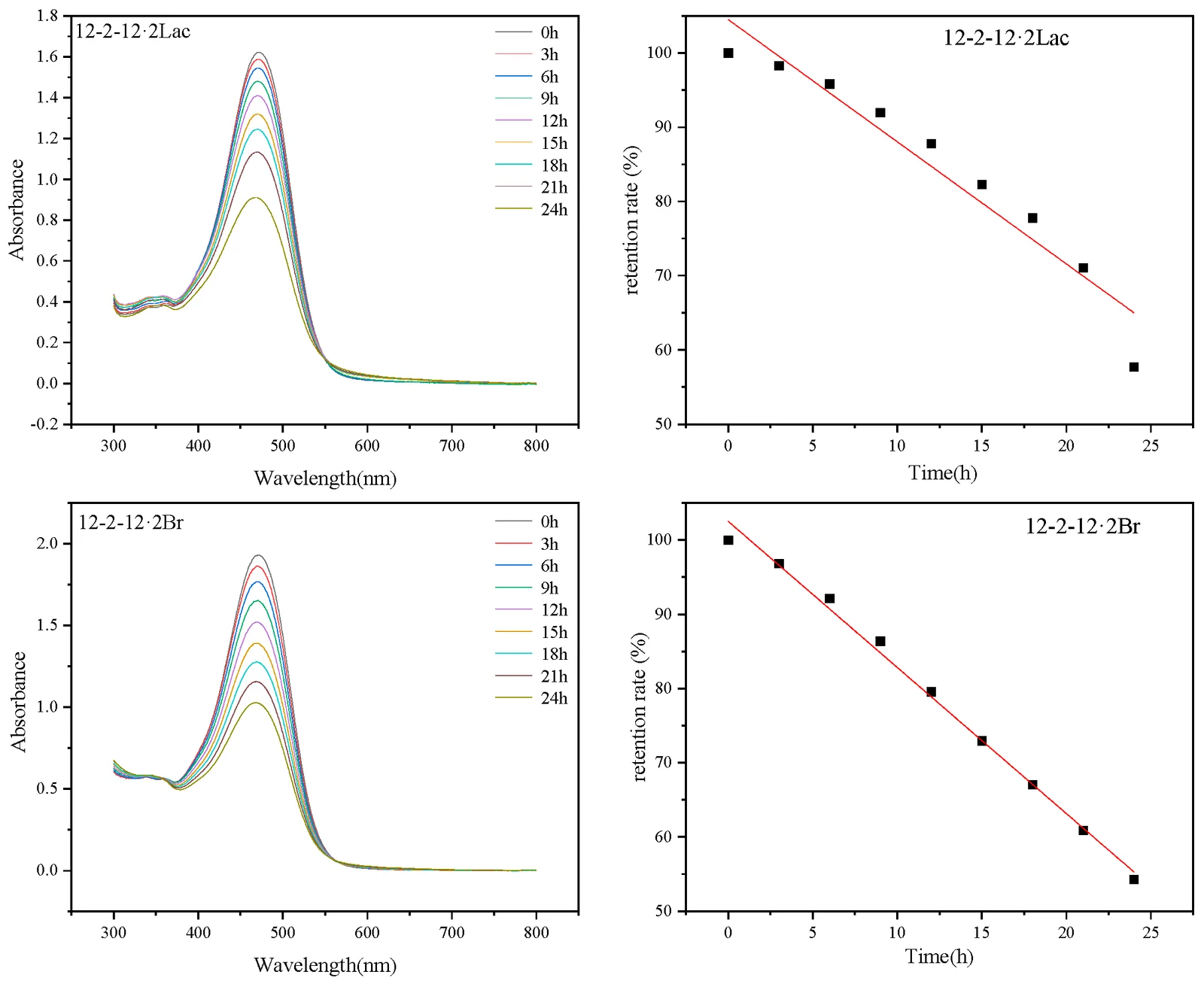
Changes in the absorption spectra and the retention rate of Cur^3−^ (40 μM) in ultrapure water and 12-2-12·2Y micelle solutions with increasing time.

Previous studies have shown that curcumin is generally stable in acidic conditions, but undergoes rapid degradation in an aqueous solution when the pH is above neutral. In this study, the degradation kinetics of Cur^3−^ were investigated in ultrapure water and 12-2-12·2Y micelle solutions (4 CMC) by recording the UV-vis absorption spectra of Cur^3−^ every 3 h for 24 h, as shown in Figure 4. The decrease in absorbance maximum at 460 nm over time was used to evaluate Cur^3−^ degradation. The retention rates of Cur^3−^ are listed in Table 4.

As shown in Table 4, after 24 h incubation, the retention rate of Cur^3−^ in ultrapure water decreases rapidly to 4.21%, whereas 54.30–82.29% Cur^3−^ remain in the 12-2-12·2Y micelles. Furthermore, the residual concentration of Cur^3−^ in ultrapure water and the 12-2-12·2Y micelles was fitted to a pseudo-zero order model [3], and the degradation rate of Cur^3−^ is also summarized in Table 4. This treatment provides an average degradation rate at a fixed initial Cur^3−^ concentration, enabling direct comparison among different surfactants micelle systems. It is used here as an empirical kinetic description rather than as a mechanistic assignment. As illustrated in Table 4, the degradation rates of Cur^3−^ in different systems decreases in the following order: H_2_O > 12-2-12·2Br > 12-2-12·2Lac > 12-2-12·2Mc > 12-2-12·2Bz > 12-2-12·2Ac > 12-2-12·2Va. This order is also generally consistent with the order based on the fluorescence quantum yield of Cur^3−^ (Table 2), which further indicates that the microenvironment of the palisade layer affects the stability of Cur^3−^. The degradation rate of Cur^3−^ in ultrapure water is 1596.50 nM/h, which is more than five times higher than that of Cur^3−^ in the 12-2-12·2Ac (337.50 nM/h) and 12-2-12·2Va (295.17 nM/h) micelles. The degradation rate of Cur^3−^ in the 12-2-12·2Br micelles is 761.67 nM/h, which, although higher than in ultrapure water, is the lowest among all micelle systems. This is consistent with the fluorescence quantum yield and Cu^2+^ quenching results: the tight adsorption of Br^−^ onto the micellar surface weakens the electrostatic attraction to Cur^3−^, preventing it from effectively entering the protective palisade region.

### 3.6. ^1^H NMR Spectra

**Figure 5:**
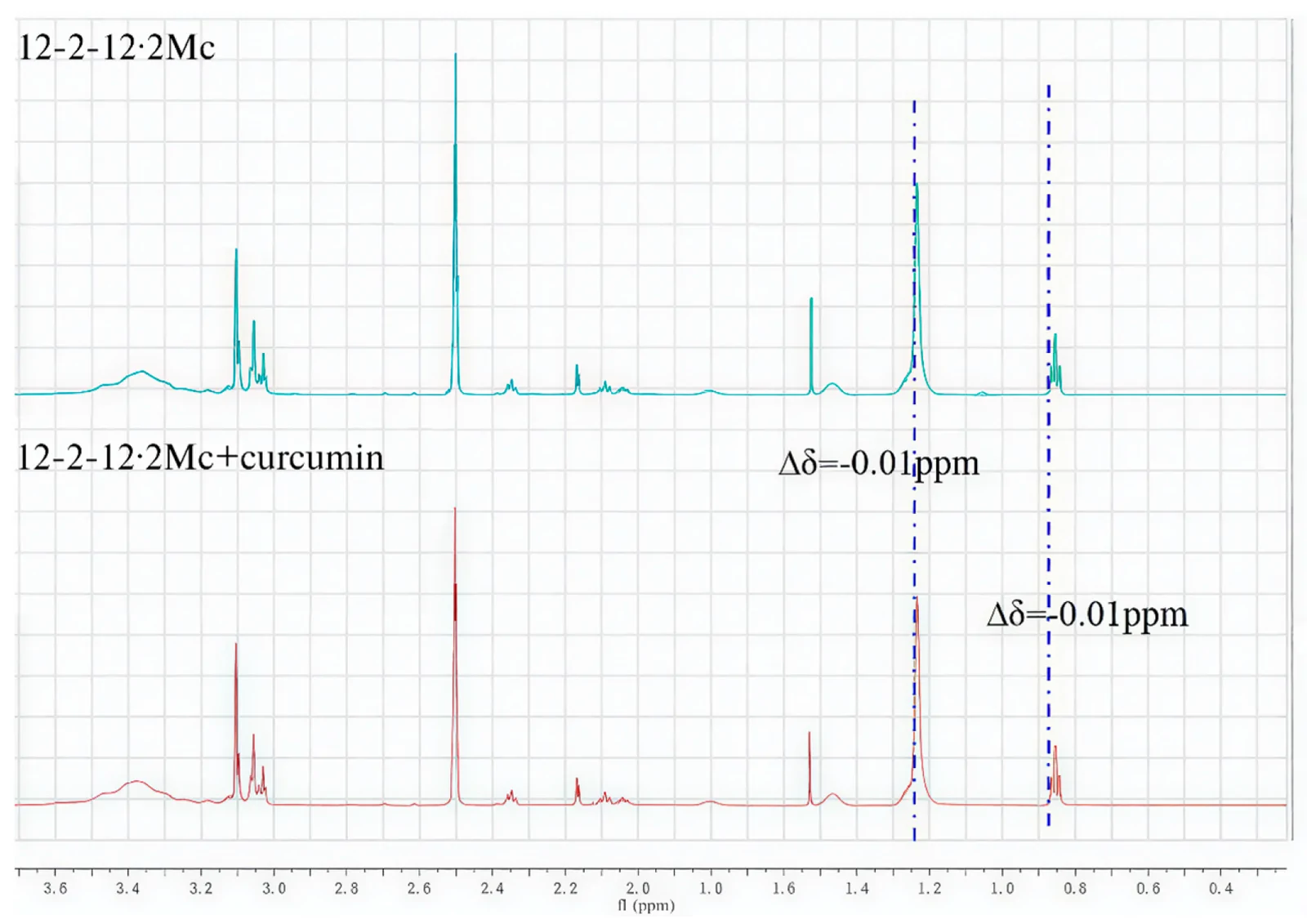
The ^1^H NMR spectra of 12-2-12·2Mc before and after the addition of curcumin.

Figure 5 shows the ^1^H NMR spectra of 12-2-12·2Mc before and after the addition of curcumin (as a representative example, since the spectra of other surfactants in the 0–3.5 ppm range are similar to these ones). As shown in Figure 5, the addition of curcumin causes the chemical shifts in the methylene and methyl protons of the alkyl chains in the 12-2-12·2Mc micelles to move upfield, i.e., to a lower chemical shift. This indicates that the addition of curcumin increases the electron density around these protons. This upfield shift is due to the formation of hydrogen bonding interactions between the electron-rich phenolic hydroxyl and methoxy groups of curcumin and the amide groups of the surfactant, as well as hydrophobic interactions between the aromatic ring system of curcumin and the alkyl chains. These non-covalent interactions alter the local magnetic environment of the palisade layer, enhancing the shielding effect on the alkyl chain protons [38]. The ^1^H NMR results in CDCl_3_ suggest the presence of specific non-covalent interactions between curcumin and the surfactant in a non-aqueous environment. However, as CDCl_3_ does not reproduce the aqueous micellar interface, these results are considered to be supporting evidence rather than direct proof of palisade-layer localization. The interfacial distribution of Cur^3−^ can therefore be inferred from the combined fluorescence, quantum yield and quenching results, and this should be verified using D_2_O-based ^1^H NMR in future studies.

### 3.7. DPPH Radical Scavenging Ability

**Figure 6:**
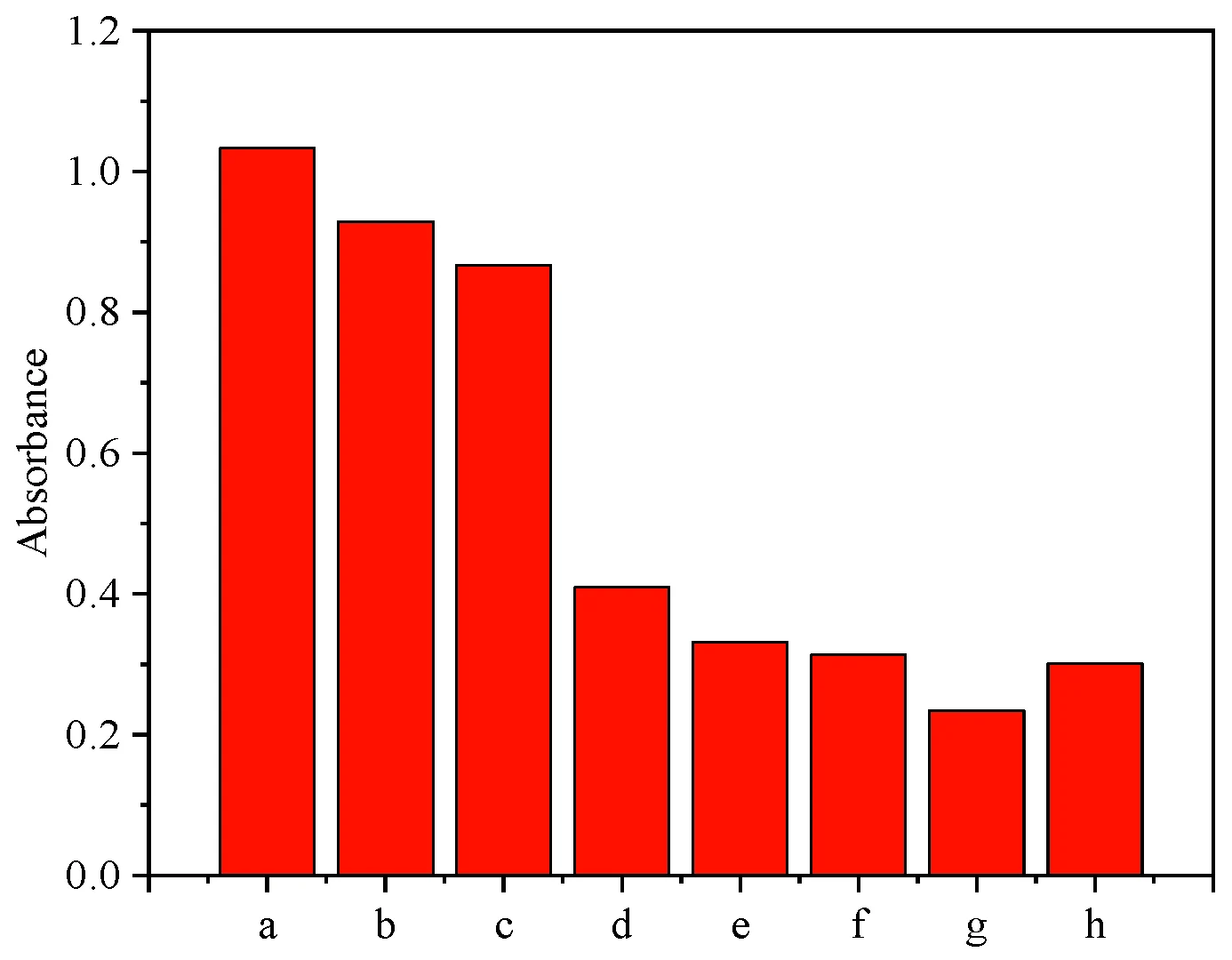
The absorbance of DPPH at 535 nm (a), as well as the absorbance in the presence of curcumin (b), and in the presence of curcumin and 12-2-12·2Mc (c), 12-2-12·2Ac (d), 12-2-12·2Va (e), 12-2-12·2Lac (f), 12-2-12·2Bz (g), 12-2-12·2Br (h).

The DPPH radical scavenging assay was used to evaluate the antioxidant activity of curcumin in the presence of 12-2-12·2Y micelles. When reduced by an antioxidant, the absorbance of DPPH at 535 nm decreases, enabling the radical scavenging capacity to be assessed quantitatively [39]. Figure 6 shows the absorbance of DPPH at 535 nm, as well as the absorbance in the presence of curcumin, and in the presence of curcumin and surfactants 12-2-12·2Y (4 CMC).

As shown in Figure 6, the DPPH solution alone exhibits an absorbance of 1.033 at 535 nm. When curcumin is added, the absorbance decreases to 0.929, indicating that curcumin possesses intrinsic radical scavenging activity. The presence of 12-2-12·2Y micelles further decreases the absorbance of DPPH, suggesting that these micelles can significantly enhance the antioxidant activity of curcumin. This enhancement arises from three factors: (I) improved solubilization and dispersion of curcumin, (II) conformational restriction of the active moieties of curcumin, and (III) interfacial enrichment of curcumin and DPPH radicals at the palisade layer of the micellar structure [40]. Additionally, when the concentration of 12-2-12·2Y is 4 CMC, the DPPH scavenging activity of curcumin in different systems follows this order: 12-2-12·2Bz > 12-2-12·2Br > 12-2-12·2Lac > 12-2-12·2Va > 12-2-12·2Ac > 12-2-12·2Mc. This order is not fully consistent with either the *K*_SV_ or fluorescence quantum yield trends, indicating that the radical scavenging efficiency may be governed by a complex interplay of interfacial charge, palisade-layer compactness and β-diketone accessibility. The highest activity of curcumin in the 12-2-12·2Bz system may be attributed to the π–π stacking of the benzoate moieties. This enhances micellar ordering and promotes an extended curcumin conformation with improved active-site accessibility. Curcumin exhibits high activity in the 12-2-12·2Br system. This may be because its electrostatic anchoring is relatively weak, giving curcumin greater conformational freedom. However, it is possible that the compact palisade layer of 12-2-12·2Va promotes the stability and fluorescence intensity of curcumin but also acts as a physical barrier to DPPH penetration. Consequently, curcumin in the 12-2-12·2Va system shows moderate activity. It is worth noting that the radical-scavenging activity of curcumin in the 12-2-12·2Mc system is relatively low. This may be due to the strong polarity and potential oxidizing properties of CH_3_CO_3_^−^. These properties may either lock down the active hydrogen of curcumin through hydrogen bonding or dipole–dipole interactions, or introduce competitive redox pathways, thereby diminishing net DPPH reduction [41].

## 4. Conclusions

In this study, the interaction between Cur^3−^ and a series of gemini quaternary ammonium surfactants with different counterion micelles were systematically investigated. Steady-state fluorescence spectroscopy and ^1^H NMR spectroscopy confirm that there is a strong electrostatic attraction between Cur^3−^ and the positively charged headgroups of 12-2-12·2Y, which causes Cur^3−^ to be preferentially distributed in the palisade layer. Cur^3−^ exhibits the highest quantum yield in the 12-2-12·2 Va micelles, which may be attributed to their highly compact and hydrophobic palisade layer, whereas the lowest yield was obtained in the 12-2-12·2Br micelles, possibly due to the strong hydration of Br^−^ and its tight adsorption onto the headgroups. DPPH radical scavenging assays demonstrate that micellar solubilization/stabilization can enhance the antioxidant activity of curcumin. Radical scavenging efficiency may be governed by the interplay between interfacial charge, palisade-layer compactness and β-diketone accessibility, revealing a trade-off between chemical stability and antioxidant activity. The counterions may influence the efficiency of radical scavenging by modulating the positioning of curcumin within the palisade layer and the accessibility of the β-diketone moiety. It seems that there is an apparent trade-off between the chemical stability and antioxidant activity of curcumin: compact palisade layers of 12-2-12·2Y favor curcumin’s stability but may hinder its radical scavenging, whereas looser interfaces of 12-2-12·2Y facilitate curcumin’s antioxidant action at the expense of its stability. In summary, counterions may regulate the positioning, fluorescence behavior, chemical stability and antioxidant activity of Cur^3−^ in the palisade layer via multiple mechanisms, including electrostatic attraction/repulsion, the balance between hydrophilic and hydrophobic forces, and steric hindrance. From a translational perspective, the present micellar systems should be regarded as mechanistic models rather than immediate food formulations. The experiments were performed at pH 13, which differs greatly from the pH of most foods and physiological systems. Furthermore, comprehensive evaluation of synthetic cationic gemini quaternary ammonium surfactants’ cytotoxicity, biodegradability and regulatory compliance is required before they can be used in food contact or oral delivery applications. Therefore, future work should focus on food-grade surfactant alternatives, neutral pH formulations, low surfactant concentrations, encapsulation efficiency and release behavior, sensory and matrix compatibility, and scalable manufacturing.

## Figures and Tables

**Table 1:** Micellization parameters of the surfactants 12-2-12·2Y.

Surfactant	CMC (mol/L)	*I*_1_/*I*_3_
12-2-12·2Br	2.23 × 10^−4^	1.21
12-2-12·2MC	2.56 × 10^−4^	1.23
12-2-12·2Ac	7.57 × 10^−5^	1.42
12-2-12·2Va	2.33 × 10^−4^	1.23
12-2-12·2Bz	8.45 × 10^−5^	1.11
12-2-12·2Lac	2.14 × 10^−4^	1.30

**Table 2:** The fluorescent quantum yields of Cur^3−^ in ultrapure water and micellar solutions.

Micelle Type	Fluorescence Quantum Yield
Ultrapure water	2.12 × 10^−4^
12-2-12·2Br	1.79 × 10^−4^
12-2-12·2Mc	1.52 × 10^−3^
12-2-12·2Ac	2.94 × 10^−4^
12-2-12·2Va	1.70 × 10^−3^
12-2-12·2Bz	1.25 × 10^−3^
12-2-12·2Lac	8.65 × 10^−4^

**Table 3:** The Stern–Volmer quenching constant values of Cur^3−^ in the solvent (V_ethanol_:V_water_ = 85:15) and 12-2-12·2Y micelle solutions.

Micelle Type	*K*_SV_	R^2^
Solvent (ethanol:water = 85:15)	0.79089	0.97774
12-2-12·2Mc	0.63856	0.99070
12-2-12·2Ac	1.65972	0.98694
12-2-12·2Va	1.19222	0.95586
12-2-12·2Lac	0.05872	0.97385
12-2-12·2Bz	1.02889	0.95044
12-2-12·2Br	0.04250	0.98484

**Table 4:** The retention and degradation rates of Cur^3−^ in ultrapure water and 12-2-12·2Y micelle solutions after 24 h incubation.

Micelle Type	Retention Rate (%)	Degradation Rate (nM/h)
Ultrapure water	4.21	1596.50
12-2-12·2Br	54.30	761.67
12-2-12·2Mc	72.81	453.17
12-2-12·2Ac	79.75	337.50
12-2-12·2Va	82.29	295.17
12-2-12·2Bz	75.20	413.33
12-2-12·2Lac	57.72	704.67

## Data Availability

The original contributions presented in this study are included in the article. Further inquiries can be directed to the corresponding authors.
